# L1 slope: an overlooked spinal parameter

**DOI:** 10.1007/s00402-024-05311-8

**Published:** 2024-04-20

**Authors:** Ahmet Celal Iplikcioglu, Hamza Karabag

**Affiliations:** 1 Department of Neurosurgery, BHT Clinic Istanbul TEMA Hospital, İstanbul, Turkey; 2 Department of Neurosurgery, Faculty of Medicine, Harran University, Şanlıurfa, Turkey

**Keywords:** Lordosis, Health-related quality of life, Supine positions, Regression analyses

## Abstract

**Objective:**

Lumbar lordosis can be divided into two parts by a horizontal line, creating the L1 slope and the sacral slope. Despite being a major spinopelvic parameter, the L1slope (L1S) is rarely reported. However, there is some evidence that L1S is a relatively constant parameter. This study aimed to analyze the L1 slope and its relationships with other spinopelvic parameters.

**Methods:**

Standing lateral lumbosacral x-ray radiographies of 76 patients with low back pain and CT scans of 116 asymptomatic subjects were evaluated for spinal and spinopelvic parameters including L1 slope (L1S). The x-ray and CT groups were divided into subgroups according to mean sacral slope (SS) or pelvic incidence (PI) values. The mean values of the spinopelvic parameters and the correlations between them were investigated and compared.

**Results:**

L1S was 19.70 and 18.15 in low SS and high SS subgroups of x-ray respectively. L1S was 7.95 and 9.36 in low and high PI subgroups of CT, respectively, and the differences were insignificant statistically. L1S was the only spinal parameter that did not change as SS or PI increased in standing and supine positions. L1S was correlated with lumbar lordosis (LL) proximal lumbar lordosis (PLL) and distal lumbar lordosis (DLL) in both x-ray and CT groups. L1S was also the strongest correlated parameter with pelvic incidence lumbar lordosis mismatch (PI-LL) mismatch in supine position.

**Conclusions:**

L1S is a relatively constant parameter and is around 16°–18° and 8°–9° in the standing and supine positions, respectively. It was significantly correlated with LL, PLL, DLL, and PI-LL. In the standing position it was nearly equal to PLL while this equality was present in low PI subgroups of CT. There is strong evidence that L1S is significantly correlated with health-related quality of life scores.

**Supplementary Information:**

The online version contains supplementary material available at 10.1007/s00402-024-05311-8.

## Introduction

The pelvis and spine must be aligned to provide balance and minimize energy consumption during activities. This is referred to as spinopelvic alignment and is evaluated according to pelvic incidence (PI), pelvic tilt (PT), and sacral slope (SS), which also affect lumbar lordosis (LL) and thoracal kyphosis curvatures [1–4]. PT and SS are positional parameters, and PI is the sum of PT and SS. PI defines the position of the sacrum within the pelvis and is the most important parameter. PI is also a unique morphological parameter in that it does not change with position. There are strong correlations between PI, PT, SS, and LL; the higher the PI, the less vertical the sacrum. This causes an increase in the LL angle [1, 3]. 

Studies have shown the spinopelvic parameters to be correlated with certain spinal pathologies including scoliosis, spondylolisthesis, disc degeneration, and adjacent segment disease [4–6]. They are also related to the severity of pain, disability, and reductions in health-related quality of life [7, 8]. Some authors have developed predictive formulas (such as PI-LL < ± 9°) that determine the ideal LL for surgical planning [9]. LL can be divided into two parts by a horizontal line, creating the L1 slope and the sacral slope. Despite being a major spinopelvic parameter that is highly correlated with LL, the L1slope (L1S) is rarely reported. However, there is some evidence that L1S is a relatively constant parameter [10–12]. The present study aimed to analyze the L1S and its relationships with other spinopelvic parameters.

## Materials and methods

### Ethics statement

This study was conducted in accordance with the tenets of the 2013 revision of the Declaration of Helsinki. It was approved by Local Ethics Committee. Informed consent was not obtained from patients because of retrospective nature of the study.

### Inclusion and exclusion criteria

This retrospective study was conducted at Harran University, Şanliurfa, Turkey. Standing lateral lumbosacral radiographies of patients with low back pain and abdominal pelvic computed tomography (CT) scans of patients with acute abdominal and urologic problems performed between June 2018 and June 2019 were reviewed. The demographic and clinical characteristics of the patients were also extracted from the medical records, including age, height, body mass index, and medical history.

In the first group (standing radiographs), patients with low back pain starting within the last three months, without motor deficit, and who were treated conservatively were included. For the second group (supine CT), we included patients who presented with acute abdominal or urological problems for which they underwent abdominal CT scans. In general, all included patients were aged between 18 and 60 years, had a body mass index of 18.5–40 kg/m^2^, and had no previous history or complaints of spinal, pelvic, hip, or lower extremity diseases, trauma, or surgery. In addition, the following radiological inclusion criteria were used for both groups:

1. Absence of transitional lumbosacral vertebrae, spondylolisthesis, or vertebral fracture.
2. Absence of radiological findings (on radiographs or CT) of degenerative spine disorder, such as the collapse of disc space, endplate sclerosis, degenerating facets, vacuum sign, or spinal stenosis.
3. Coronal Cobb angle < 10°.
4. In the X-ray group, LL and SS values should be within the range of values previously reported for healthy populations (20°–80° for LL and 13°–65° for SS).
5. In the CT group, patients with PT values < 20° and PI–LL values < 11° were included. However, those with PI–LL values between 10°–20° were included if their PT values were < 20°.

Accordingly, 76 patients were included in the first group and 116 patients in the second group.

### Measurements

The standing lateral lumbosacral plain x-ray radiography was performed on a XGEOGC80 X-ray instrument (Samsung, Korea). CT scans of all participants were performed on a Revolution GSI 256 MSCT (General Electric Healthcare, USA) with patients lying on their backs with their knees flexed. The measurements were performed on a Clear Canvas Workstation (Synaptive Medical, Canada) using Surgimap Software (Nemaris Inc. New York, USA). SS, LL, L1S, proximal (PLL), and distal lordosis (DLL) were measured on standing lumbosacral radiographies while PI, PT, SS, LL, L1S, proximal (PLL), and distal lordosis (DLL) were measured on CT. PI and PT could not be measured on x-ray because of poor visualization of femur heads in most of the cases. All measurements were performed twice by two experienced spinal surgeons. The time interval between measurements was > 2 weeks.

### Parameters

**Fig. 1:**
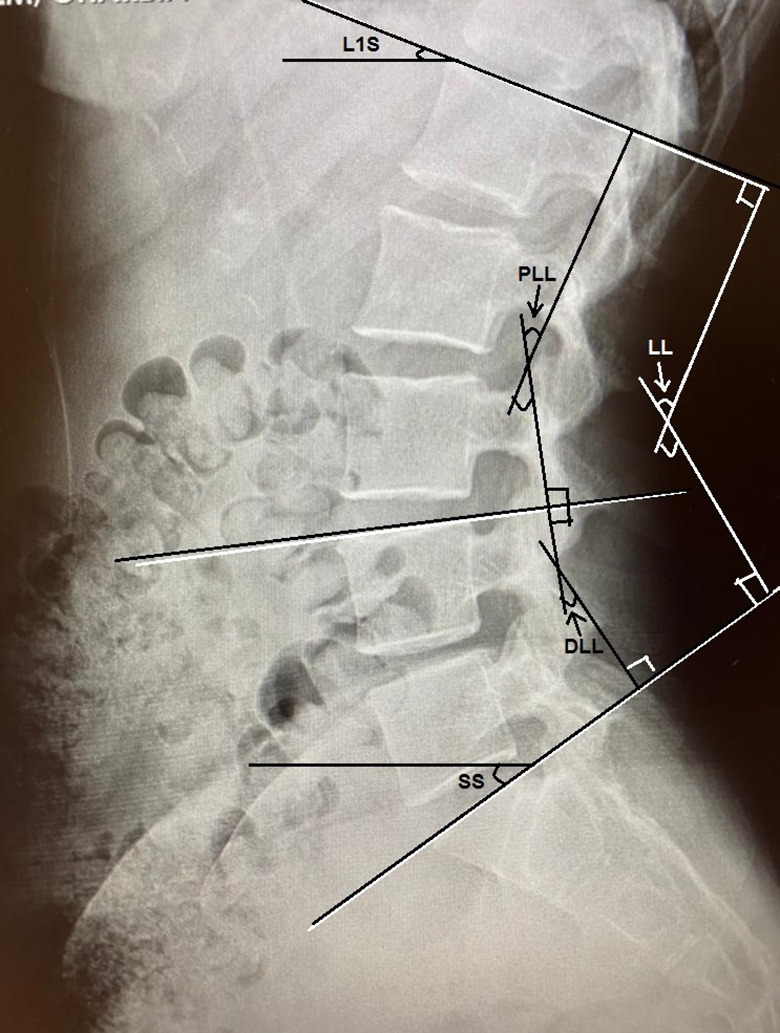
Spinal parameters measured on lateral standing lumbosacral radiography. SS: Sacral Slope, L1S: L1 Slope, LL: Lumbar Lordosis, PLL: Proximal Lumbar Lordosis, DLL: Distal Lumbar Lordosis

**Fig. 2:**
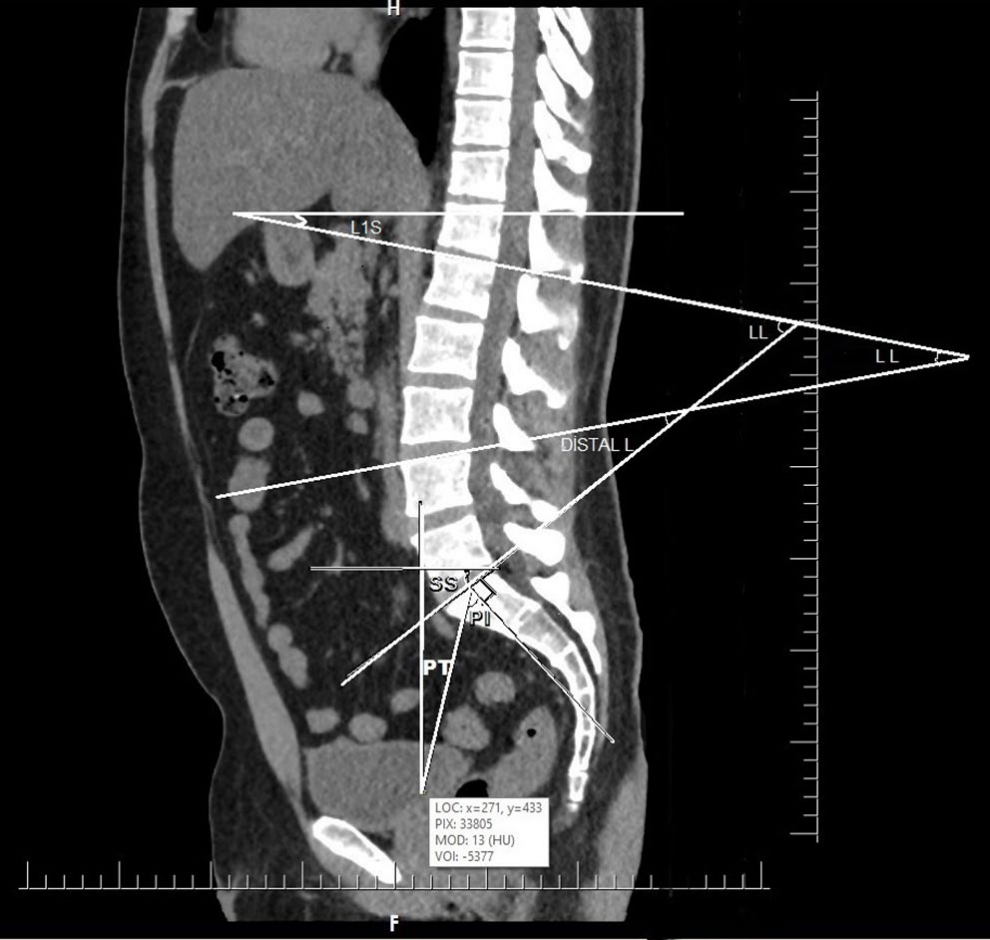
Spinopelvic parameters on a midsagittal slice of lumbosacral computed tomography. PI: Pelvic Incidence, PT: Pelvic Tilt, SS: Sacral Slope, L1S: L1 Slope, LL: Lumbar Lordosis, PLL: Proximal Lumbar Lordosis, DLL: Distal Lumbar Lordosis

- PI: the angle between a line perpendicular to the superior sacral endplate at its midpoint and a line connecting this point to the midpoint of the axis of the femoral heads.
- PT: the angle between a line from the midpoint of the sacral endplate to the midpoint of the femoral head axis and a vertical plumb line.
- SS: the angle between a line parallel to the endplate of the sacrum and a horizontal line.
- LL: the angle between a line through the superior endplate of the L1 vertebra and a line through the superior endplate of the S1 vertebra.
- L1S: the angle between the upper endplate and a horizontal line.
- PLL: the angle between the superior endplates of the L1 and L4 vertebra.
- DLL: The angle between the superior endplates of the L4 and S1 vertebrae. (Figures 1 and 2).
- PI − LL: the PI value minus the LL value.

The measurement of PI on CT has been previously described [13]. First, the coordinates of the centers of both femur heads were found. The average of these coordinates was taken as the coordinate for the hip axis in a midsagittal slice. PI and PT were measured using this reference point.

### Statistics

The interclass correlation coefficient (ICC) was used to assess intra- and interobserver reliability. Values of 0.60–0.74 and 0.75–1.00 were considered good and excellent, respectively. The mean and standard deviation of each parameter was calculated. T-tests were used to compare parameters between the low- and high PI groups. The Pearson correlation coefficient was performed to determine correlations between the parameters. The level of statistical significance was set at *p* < 0.05. Linear regression analysis was performed for parameter prediction.

## Results

In the x-ray group there were 76 patients (36 males and40 females) with an average age of 36.74 + 13.11. The CT group included 116 subjects (54 males and 62 females), with a mean age of 41.79 ± 9.98. The intra- and interobserver ICC values for all parameters were excellent (0.88–0.94). The mean values for all of the spinopelvic and spinal parameters of the cohort are shown in Tables 1 and 2. The mean value of L1S was 18.93 + 8.15 and 8.21 + 4.33 on x-ray and CT respectively. In both groups, there was no statistically significant difference between the mean L1S values for male and female patients (X-ray: males = 18.43°, females = 19.23°; CT: males = 7.7°, females = 8.8°).

**Table 1:** Mean Values of Spinal Radiologic Parameters Measured on Standing Lumbosacral radiographies

SS	34.92 ± 8.02^o^
L1S	18.93 ± 6.55^o^
LL	53.85 ± 11.13^o^
PLL	18.888 ± 7.72^o^
DLL	34.97 ± 8.27^o^

**Table 2:** Mean Values of Spinopelvic Radiologic Parameters Measured on Supine CT

PI	48.87 ± 9.97^o^
PT	8.82 ± 4.89^o^
SS	39.98 ± 8.13^o^
LL	48.19 ± 11.09^o^
PLL	11.67 ± 7.02^o^
DLL	36.54 ± 7.41^o^
PI-LL	0.70 ± 6.80^o^
L1S	8.21±4.37^o^

The x-ray and CT groups were divided into two subgroups according to their mean SS or PI values. Those with SS values higher than the mean SS were designated as the high SS subgroup and those with SS values lower than the mean were in the low SS subgroup for x-ray. In CT group, those with PI values higher than the mean PI were designated as the high PI subgroup and those with PI values lower than the mean were in the low PI subgroup. All of the mean spinopelvic parameters were significantly different between these groups, except L1S (Tables 3 and 4). The mean L1S was 19.70 and 18.15 in the low and high SS subgroups respectively for x-ray. For CT, It was 7.95 and 9.36 in the low PI and high PI groups, respectively, and there was no significant difference between them. In the x-ray group and the CT subgroup with low PI value, the mean PLL was nearly equal to the mean L1S, suggesting that the L4 upper endplates of the patients were nearly horizontal.

**Table 3:** Comparison of Mean Values of Spinal Parameters From Low SS and High SS Groups of Standing Lumbosacral Radiographies

	Low SS (36)	High SS (40)	P Values	Significance
SS	27.82 ± 6.13^o^	41.44 ± 10.12^o^	<0.0001	S
L1S	19.70 ± 7.23^o^	18.15 ± 6.33^o^	>0.05	NS
LL	47.52 ± 10.22^o^	59.59 ± 14.18^o^	<0.0001	S
PLL	16.72 ± 7.55^o^	20.82 ± 8.20^o^	0.00715	S
DLL	30.8 ± 8.07^o^	38.79 ± 8.85^o^	0.0204	S

S: Significance, NS: Nonsignificance

**Table 4:** Comparison of mean values of spinopelvic parameters from low PI and high PI groups of supine CT

	Low PI (56)	High PI (60)	P Values	Significance
PI	40.59 ± 5.92^o^	56.09 ± 6.55^o^	<0.0001	S
PT	6.89 ± 4.36^o^	10.07 ± 4.70^o^	<0.02	S
SS	33.70 ± 5.82^o^	45.02 ± 6.05^o^	<0.0001	S
LL	40.64 ± 8.33^o^	53.06 ± 8,86^o^	<0.0001	S
PI-LL	-0.05 ± 7,22^o^	1.39 ± 6.45^o^	<0.021	S
L1S	6.94 ± 4.12^o^	7.58 ± 4.55^o^	>0.05	NS
PLL	7.62 ± 4.39^o^	15.15 ± 7.08^o^	<0.05	S
DLL	33.02 ± 6.3^o^	38.45 ± 7.03^o^	<0.05	S

S: Significance, NS: Nonsignificance

### Correlations

The results of the Pearson correlation analysis of the spinal and spinopelvic parameters are shown in Tables 5 and 6. There was no statistically significant correlation between L1S and the patient’s age (X-ray: *r* = − 0.155, *p* = 0.181; CT: *r* = 0.111, *p* = 0.256).

The correlations between PI, PT, SS, and LL in the total cohort were in the agreement with those found in previous studies. L1S was correlated with LL (r:0.630 for x-ray and r: 0.492 for CT), PLL (r: 0.454 for x-ray and r: 0.422 for CT), DLL (r:0.297 for x-ray and r: 0.293 for CT). L1S was the parameter with the strongest correlation with PI-LL mismatch (r: 0.749) Table 7. Linear regression analysis disclosed the following formulas:

-  For standing x-ray, LL:37.59 + 0.926 L1S.
-  For CT, PT: 0.2317PI—1.015
-  LL: 0.8276PI + 6.5835
-  PI-LL: PT-L1S, L1S: PT-(PI-LL)
-  L1S: 0.0593PI + 5.57 with an average PI value of 45°–50° L1S was nearly 8°–8.5°.

The predictive formula for PI-LL mismatch using L1S was established as:

-  PI-LL: 8.6529-0.9109L1S.

**Table 5:** Correlation of L1S with age and other spinal parameters on standing X-ray

		Coefficient	P Value	Significance
L1S	Age	r: -0.155	0.181	NS
L1S	SS	r: -0.086	0.629	NS
L1S	LL	r: 0.735	0.00001	S
L1S	PLL	r: 0.454	0.0048	S
L1S	DLL	r: 0.630	0.00002	S

S: Significance, NS: Nonsignificance

**Table 6:** Correlation of L1S with age and other spinopelvic parameters on supine CT

		Coefficient	P Value	Significance
L1S	Age	r: 0.111	0.256	NS
L1S	PI	r: -0.009	>0.05	NS
L1S	PT	r: -0.033	>0.05	NS
L1S	SS	r: -0.068	>0.05	NS
L1S	LL	r: 0.493	<0.001	S
L1S	PLL	r: 0.422	<0.001	S
L1S	DLL	r: 0.294	<0.01	S
L1S	PI-LL	r: -0.749	<0.0001	S

S: Significance, NS: Nonsignificance

**Table 7:** Correlation between (PI-LL) mismatch and other spinopelvic parameters

		Coefficient	P Value	Significance
PI-LL	PI	r: 0.292	<0.05	S
PI-LL	PT	r: 0.720	<0.0001	S
PI-LL	SS	r: 0.03	>0.05	NS
PI-LL	LL	r: -0.386	<0.01	S
PI-LL	L1S	r: -0.749	<0.0001	S

S: Significance, NS: Nonsignificance

## Discussion

According to Roussoly, LL is measured between the inflection point and the upper S1 endplate [14]. The inflection point is the location that determines the border of LL and thoracic kyphosis. This angle may contain more than five vertebral bodies and can be divided by a horizontal line through the apex of LL. The upper arc is constant at 15°–17°, while the lower arc is the SS [10, 14]. In most cases, the inflection point is around the L1 body. Otherwise, the angle of the upper arc differs by less than 1° from the L1S so resembles the L1S. Hey et al. found L1 to be the only vertebrae with a similar vertebral slope (14.4°–16.3°) across Roussoly curve types [11]. 

The two formulas that allow us to determine the mean L1S from clinical and radiological studies are:

L1S = LL-SS.

PI-LL = PT + SS-(L1S + SS) = PT-L1S.

L1S: PT-(PI-LL).

In a systematic review of spinopelvic parameters in asymptomatic patients, (2926 patients from 17 studies), the mean L1S was found to be 16.1° [15]. In a series by Bourret et al., the mean L1S were 17.3°, 18.3°, and 17.6° in 486 asymptomatic patients with low, normal, and high PI, respectively [16]. Pesenti et al. found mean L1S of 20.3°, 20.6°, and 18.8° in low, normal, and high PI groups, respectively [17]. Bourret et al. also reported the following equations [16]:

LL = 28.4291 + 0.5578PI.

PT = − 10.2499 + 0.4374PI.

PI-LL = − 28.4291 + 0.4422 PI From this, we can calculate the mean S1 slope as follows:

L1S = 18.18°−0.005PI.

Similar results were obtained from regression analyses by Legaye and Duval-Beaupere, Le Huec et al., and Hasegawa et al. [18–20] A PI of 0.005 is negligible because of its very low value. This finding suggests that L1S is relatively constant at around 18°.

In our study for CT, L1S was 0.06 (PI) + 5.57°, with an average PI value of 45°–50° L1S was nearly 8°–8.5°. The difference was due to the supine position used for our CT studies. In the supine position, the LL decreases by 3°–5°, while the SS increases by 3°–5°. So, L1S decreases by 8°–10°. Therefore, based on our results, the mean L1S in a standing position would be ~ 18°.This finding is also supported by the study of Hariyama et al. concluded that positional change in lordosis occurred primarily through the upper lumbar segments [21]. In our study, the PI and LL values for all groups (cumulative, low, and high PI) were nearly equal so the values of PI-LL were − 1.05°–1.4°. This finding is in accord with those from other studies performed with patients in the supine position [22, 23]. According to LL prediction formulas such as those described by Le Huec et al. and Hasegawa et al. [19], LL = 0.50PI + 28. Thus, LL is 3°–4° greater than PI when PI is less than 60°. This means that, in most cases, LL will decrease and become closer to PI when the patient is in the supine position. [19]

These findings also support the hypothesis that L1S is not significantly correlated with PI. We found that L1S was correlated with LL, PLL, DLL, and PI-LL. These relationships with other spinopelvic parameters are in line with those previously reported [12]. L1S and PT were the parameters with the strongest correlations with PI-LL; although, the correlation between L1S and PI-LL was negative. PI-LL is an important spinal parameter and has been found to modify adult spinal deformity (ASD) classifications [24]. 

We also found L1S to be correlated with PLL and DLL. In the x-ray group and low PI subgroup of CT, the mean PLL value was equal to the mean L1S value so, SS = DLL. This finding is in agreement with those of Hey et al., who found an L4 vertebral slope of ~ 0° in Roussoly groups 1 and 2 (low PI) [11]. However, in the Pesenti et al. cohort, DLL was nearly equal to SS in their average PI group (PI: 45°–65°) [17], Therefore, L1S nearly equals PLL in patients with low PIs.

There were several limitations in our study. First, absence of PI and PT parameters in x-ray group. Although lateral lumbar radiography is a good alternative to whole spine radiography for the measurement of spinopelvic parameters [25], in our series poor identification of hip centers was present in most of the cases so we could not measure PI and PT values. However, SS is a highly correlated parameter with PI and high SS and low SS groups could represent high PI and low PI groups respectively. Another associated problem was not able to use spinal modifiers in the X-ray group for the detection of deformity patients. Adult spinal deformity is usually associated with spinal degenerative disorders and a loss of lordosis. Such marked spinal degenerative changes are commonly seen in the elderly population; therefore, these patients were excluded from our study.

Another limitation was the mismatch between the X-ray and CT groups. We evaluated L1S and its relationships with other parameters in two different positions (standing X-rays and supine CT). Because of the retrospective nature of the study, patients in the X-ray group were symptomatic while those in the CT groups were asymptomatic. To minimize this effect, we ensured that the patients in the group X-ray were only mildly symptomatic without motor deficits; also, they were relatively young. Therefore, the mean L1S, LL, and SS values reported for this group were very close to that of healthy subjects and can be accepted as an asymptomatic group. On the other hand, we thought that evaluation of spinopelvic parameters in supine position strengthened our study, because the importance of supine imaging tools has been increasing for several reasons [26–28]. First, the correlation between the spinopelvic parameters are strongest in the supine position and lumbosacral sagittal alingment in the supine position is appromixately equal to that in the prone position which is actually intraoperative position, so supine images can be helpful for the surgical planing [26]. Second, the difference between LL values measured in supine and standing position was found correlated with postoperative outcome in the patients with adult spinal deformity [27, 28]. We believe that supine imaging will be utilized more commonly in the future for the diagnosis and the treatment of spinal disease.

## Conclusion

L1S is a relatively constant parameter and is around 18–19° and 8°–9° in the standing and supine positions, respectively. It is significantly correlated with LL, PLL, DLL, and PI-LL. In those with low PI values, L1S equals PLL. L1S is also the parameter with the strongest correlation with PI-LL and it is equal to the difference between two important modifiers of SRS-Schwab ASD classification (PT, PI-LL). However, more studies are needed to corroborate these results.

### Electronic supplementary material

Below is the link to the electronic supplementary material.

Supplementary Material 1
