# Supplementary material for: L1 slope: an overlooked spinal parameter
Source: Arch Orthop Trauma Surg. 2024 Apr 20;144(5):2077–83. doi: 10.1007/s00402-024-05311-8 (PMC11093775; doi:10.1007/s00402-024-05311-8)
Supplement: Supplementary file 1 — Supplementary Material 1 [file 402_2024_5311_MOESM1_ESM.docx]

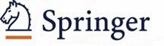


Conflict of Interest Disclosure Form

It is the policy of the journal *Pediatric Radiology* to ensure balance, independence, objectivity, and scientific rigor in the Journal. All authors are expected to disclose to the readers any real or apparent conflict(s) of interest that may have a direct bearing on the subject matter of the article. This pertains to relationships with pharmaceutical companies, biomedical device manufacturers or other corporations whose products or services may be related to the subject matter of the article or who have sponsored the study.

The intent of the policy is not to prevent authors with a potential conflict of interest from publication. It is merely intended that any potential conflict should be identified openly so that the readers may form their own judgement about the article with the full disclosure of the facts. It is for the readers to determine whether the authors’ outside interest may reflect a possible bias in either the exposition of the conclusions presented.

All authors will complete and submit this form when submitting a manuscript to *Pediatric Radiology*. **We prefer disclosures and signatures from all authors on one form.** If this is not possible, however, separate forms will be accepted.

Article title: **L1 SLOPE: AN OVERLOOKED SPINAL PARAMETER**

*Please note that a conflict of interest statement is published with each paper and must be inserted in your text document right before the reference list.*

I/we certify that there is no actual or potential conflict of interest in relation to this article.

X

(Please print names)

| (1^st^ author): Ahmet Celal İplikçioğlu | | Signature: | Date:14.01.2023 |
| --- | --- | --- | --- |
| (2^nd^ author): Hamza Karabağ | | Signature: | Date:14.01.2023 |
|  |  |  |  |
|  | |  |  |
|  | |  |  |
|  | |  |  |
|  | |  |  |
|  | |  |  |
|  |  |  |  |

Article title: **L1 SLOPE: AN OVERLOOKED SPINAL PARAMETER**

🞏 I/we disclose the following potential conflicts (describe financial interest/arrangement with one or more organizations that could be perceived as a real or apparent conflict of interest in the context of the subject of this article):

Conflict: **This article does not have a financial conflict of interest with one or more organizations.**

| Print Name(s): Ahmet Celal İplikçioğlu | Signature(s): | Date:14.01.2023 |
| --- | --- | --- |
|  |  |  |
|  |  |  |
| Print Name(s): ): Hamza Karabağ | Signature(s): | Date:14.01.2023 |
| Conflict: |  |  |
|  |  |  |
| Print Name(s): | Signature(s): | Date: |
| Conflict: |  |  |
|  |  |  |
| Print Name(s): | Signature(s): | Date: |

(Use additional pages, if needed.)

Please upload this form together with your manuscript at online submission. All authors must use this form to disclose conflicts of interest or to attest to no conflicts of interest before your manuscript will be further considered.
